# Viral pathogens of acute gastroenteritis in Egyptian children: role of the parechovirus

**DOI:** 10.1186/s12879-022-07562-5

**Published:** 2022-06-29

**Authors:** Mervat El-Sayed Mashaly, Nashwa M. Alkasaby, Asmaa Bakr, Maysaa El Sayed Zaki, Karim Montasser

**Affiliations:** 1 Clinical Pathology Department, Faculty of Medicine, Mansoura University, Mansoura, Egypt; 2 Medical Microbiology and Immunology Department, Faculty of Medicine, Mansoura University, Mansoura, Egypt; 3 Pediatric Department, Faculty of Medicine, Mansoura University, Mansoura, Egypt; 4 Clinical Pathology Department, Helwn Faculty of Medicine, Helwn University, Cairo, Egypt

**Keywords:** Rotavirus, Astrovirus, Norovirus, HPeV, Gastroenteritis

## Abstract

**Background and aim:**

Human parechovirus (HPeV) has emerged as a pathogen associated with acute gastroenteritis (AGE).

**Aim:**

To detect the presence of HPeV in the stool samples from Egyptian children with AGE seeking care and the possibility of its co-infection with other enteric viruses.

**Methodology:**

One hundred stool samples were collected from children attending Mansoura University Children's Hospital with AGE. HPeV and astrovirus were detected by reverse transcriptase-polymerase chain reaction (RT-PCR). At the same time, detection of rotavirus antigen and norovirus was achieved by enzyme-linked immunosorbent assay and rapid immunochromatographic method, respectively.

**Results:**

The most frequently detected virus was rotavirus (39%), followed by norovirus (27%), HPeV (19%), and astrovirus (12%). Interestingly, the single infection with HPeV was 5%. Among the 19 HPeV positive samples, the co-infection of HPeV with other enteric viruses was detected in 9(43.9%) for rotavirus, 7(36.8%) for norovirus, 2(10.5%) for astrovirus, in 3(15.8%) for rotavirus and norovirus and 1(5.3%) for norovirus and astrovirus. Regarding the clinical presentation, there was no significant difference between children infected with HPeV alone and those infected with viruses other than HPeV alone; fever (p = 0.3), vomiting (p = 0.12), abdominal pain (p = 0.12), and grades of severity (P = 0.82). HPeV alone infected children were of mild severity (60%), and their main presenting symptom was fever (60%).

**Conclusions:**

Detection of HPeV as a single viral pathogen in the stool of some children with AGE showed that this virus could be a causative agent of AGE in Egyptian children. Therefore, HPeV could be included as one of the viruses screened for AGE diagnosis in children in Egypt.

## Introduction

Acute gastroenteritis (AGE) is a global infectious disease in children with high morbidity and mortality [1]. The pathogens associated with this infection include bacteria, parasites, and viruses [2]. AGE-associated pathogens include rotavirus A, norovirus, sapovirus, astrovirus, adenovirus, enterovirus, and human bocavirus [3, 4]. AGE in children has been associated with HPeV in a previous study [5].

Human parechoviruses are related to enteroviruses in the Picornaviridae family.

HPeVes are non-enveloped RNA viruses. There are six species within the HPeV genus classified from A to F [1]. The species that affect humans is the HPeV A [6]. The infection caused by HPeV A ranges from asymptomatic infection to severe infections such as meningitis and sepsis [7]. According to the genotype, there are 19 genotypes of HPeV with a different spectrum of infections [8]. HPeV genotype three is associated with sepsis and central nervous system infection in neonates and young infants [9]. The infection with HPeV associated with AGE is expected in early infancy and children below two years. The infection occurs through fecal–oral and respiratory routes [10]. The average duration of shedding of HPeV in the stool is more than 50 days [11].

The gold standard technique for laboratory diagnosis of HPeV is the cultivation of the virus and serotyping [12, 13]. However, this method is low sensitivity and requires a long time [14, 15]. Currently, the molecular method of reverse transcriptase-polymerase chain reaction (RT-PCR) has been used with good sensitivity [1].

In Egypt, various studies investigated various viruses such as rotavirus, astrovirus, and norovirus as an etiological pathogen in children with AGE [16], [17]. However, there are no reports about HPeV association with AGE in Egyptian children. Therefore, the present study aimed to detect the presence of HPeV in the stool samples from children with AGE by reverse transcriptase-polymerase chain reaction (RT-PCR) as well as other enteric viruses; astrovirus by R.T.- PCR; rotavirus by enzyme-linked immunosorbent assay (ELISA) and norovirus by the rapid immunochromatographic method.

## Material and method

This cross-sectional study included 100 stool samples from children with AGE. The children included had the inclusion criteria. The sample size was calculated by the non-probability sampling method as the sample size depends upon the time of the study.

The children were recruited from Mansoura University Children's Hospital from November 2021 to May 2022. The included children had AGE with age below five years. The diarrhea was defined as ⩾three episodes within 24 h with or without vomiting, fever, and abdominal pain. The severity of AGE was assessed according to the Vesikari classification [18]. The stool samples were included after excluding the common bacterial pathogens and parasitic infestation by microbiological culture and microscopic examination. The study was approved by the Mansoura Faculty of Medicine Ethical Committee (R.21.11.1536). The study was performed according to the declaration of Helsinki.

### Laboratory investigations

Each child's stool sample was obtained in a clean container and transported to the laboratory within 30 min. All containers were autoclaved after overnight treatment with diethylpyrocarbonate (1%) to inactivate the RNase. The stool sample was divided into two aliquots, one for detection of rotavirus by enzyme-linked immunosorbent assay (ELISA) and norovirus by rapid qualitative test immunochromatographic test (The RIDA®QUICK- R-Biopharm AG -An der Neuen Bergstraße 1764297 Darmstadt, Germany). RT-PCR used the second aliquot for viral nucleic acid extraction to detect astrovirus and HPeV.

### Detection of norovirus by immunochromatographic method

This method is rapid for the qualitative detection of norovirus genogroup 1 (G.I.) and genogroup 2 (GII) in stool samples. The test was performed according to the manufacturer's manual. The sample was added to a dilution buffer supplied by the kit and thoroughly mixed. The sample was allowed to stand for five minutes to yield clear supernatant, then 150 µl were added to the sample well in the test cassette. The result was available after 15 min.

### Detection of astrovirus and HPeV by RT-PCR

#### RNA extraction from stool sample

RNA was extracted from the stool sample immediately after delivery of the stool to the laboratory and then kept frozen at − 80 °C until amplification procedures. The fecal specimens were diluted (30%) with 0.01 M phosphate-buffered saline (pH = 7.2) and then centrifuged at 10,000*g* for 15 min. Supernatants were then used to extract the viral nucleic acid using a QIAamp viral RNA mini kit (Qiagen, Hilden, Germany).

#### Reverse transcription for extracted RNA

At first, the extracted RNA was incubated at 70 °C for 5 min for denaturation, then put on ice for 2 min. Then incubation was performed with reverse transcriptase enzyme for one hour at 42 °C by the use of Superscript One-Step RT-PCR with Platinum® Taq kit (Invitrogen, Carlsbad, CA, USA). The reverse transcription was carried out on the extracted RNA for molecular detection of astrovirus and HPeV.

#### PCR for astrovirus

The used primers for the detection of astrovirus are listed in Table 1. The amplification procedure was previously described [10]. It included 40 cycles of amplification (94 °C/30 s, 50 °C/30 s, 72 °C/1 min), then extension at 72 °C for one minute. The PCR product was analyzed by electrophoresis in 1.5% agarose gel stained with ethidium bromide to visualize the amplified DNA. Sterile distilled water was used as a negative control.

**Table 1:** Primers used to detect astrovirus and HPeV and the amplicon size

Target gene	Primer sequences	bp
**Astrovirus** ORF2	**F:** 5'CAACTCAGGAAACAGGGTGT3' **R:** 5'TCAGATGCATTGTCATTGGT3'	449
**(HPeV)** VP1	**F:** 5′-CCAAAATTCRTGGGGTTC-3′ **R:** 5′-AAACCYCTRTCTAAATAWGC-3′	760

*VP1* viral protein 1, *bp* base pair

#### PCR for HPeV

The primers used to detect HPeV in the stool sample were primers specific for the viral protein 1 (VP1) gene. The amplification was performed by the ready-to-use Qiagen mixture (catalog No.-201443, Qiagen-19300 Germantown Rd Germantown MD 20874 USA). The primer's sequences are listed in Table 1.

With a total volume of 50-μL with 0.5 mmol/L of the forward primer (VP1-F1) and 0.5 mmol/L of the reverse primer (VP1-R1). The amplification reaction consisted of cDNA synthesis at 45 °C for 30 min pr-denaturation at 95 °C for 2 min, followed by PCR amplification for 45 cycles at 95 °C for 30 s, at 50 °C for 30 s, and 72 °C for 1 min; followed by a final extension step at 72 °C for 7 min. PCR products were analyzed by electrophoresis on a 1.5% agarose gel [19].

### Statistical analysis

The data was analyzed by SPSS 22 program. The categorical data were expressed as numbers and percentages and compared by the Chi-square test. A p-value less than (0.05) was considered significant.

## Result

### Demographic and clinical characteristics of the children understudy

The study included 100 children with AGE with a minimum age of 1.0 months and a maximum age of 59.0 months. The children were mainly from rural regions (65%). In addition to diarrhea, they were presented with fever (38%), vomiting (32%), and abdominal pain (38%). The severity, according to Vesikari classification, was mild in (50%), moderate in (33%), and severe in (17%) of the studied children.

### Virological investigations of the stool samples

The viral study of the collected stool samples revealed that the most common virus was rotavirus (39%), followed by norovirus (27%), HPeV (19%), and astrovirus (12%) (Table 2).

**Table 2:** Frequency of the investigated viruses in 100 stool samples

Virus	Positive	Negative
	No. (%)	No. (%)
Rotavirus	39 (39)	51 (51)
Norovirus	27 (27)	73 (73)
Astrovirus	12 (12)	88 (88)
HPeV	19 (19)	81 (81)

HPeV was detected in the examined stool sample either as a single pathogen (5/100) or associated with the other investigated viruses (14/100). Out of the 19 detected HPeV, 9 (47.4%) were associated only with rotavirus, 7 (36.8%) were associated only with norovirus, 2 ((10.5%) were associated only with astrovirus, 3 (15.8%) were associated with both norovirus and rotavirus, and 1(5.3%) was associated with both norovirus and astrovirus. There was a statistically significant association between rotavirus, norovirus, and parechovirus (p = 0.001) (Table 3).

**Table 3:** Association of HPeV with rotavirus, norovirus, and astrovirus among the studied 100 children with AGE

	HPV positive patients		P-value
	No	%	
Rotavirus	9	47.4	0.41
Astrovirus	2	10.5	0.83
Norovirus	7	36.8	0.28
Rotavirus and norovirus	3	15.8	0.001
Norovirus and astrovirus	1	5.3%	0.19

The Chi-square test calculated P

There was no statistically significant difference in the epidemiological and clinical data between children with AGE due to HPeV alone and those with AGE due to viruses other than HPeV alone. However, it was observed that children with AGE caused by HPeV alone were from rural areas (60%), presented mainly with fever (60%), and the majority of them were of mild severity (60%) (Table 4).

**Table 4:** Comparison of the demographic and clinical data between children with HPeV as a single detected virus and children with other viruses

	Viruses causing AGE No (%)			p-value	OR	95% CI
	Total (n = 100)	HPeV alone (n = 5)	Other than HPeV alone )n = 95)			
Sex				0.31	3.03	0.32–28.2
Male	58	4 (80)	54 (56.8)			
Female	42	1 (20)	41 (43.2)			
Abdominal pain	83	2 (40)	36 (37.9))	0.92	1.09	0.17–6.9
Fever	38	3 (60)	35 (36.8)	0.3	2.6	0.0.41–16.1
Vomiting	32	0	32 (33.7)	0.12	1.08	1–1.15
Residence				0.81	0.79	0.13–5.2
Rural	65	3 (60)	62 (65.2)			
Urban	35	2 (40)	33 (34.7)			
Severity				0.82		
Mild	50	3 (60)	47 (49.5)			
Moderate	33	1 (20)	32 (33.7)			
Severe	17	1 (20)	16 (16.8)			

## Discussion

Acute gastroenteritis (AGE) in children below five years represents a global health problem [1]. There is an urgent need to monitor the prevalence of various viruses implicated in AGE to control this clinical condition.

The present study included 100 children with fever, abdominal pain, and vomiting, besides diarrhea as clinical manifestations. This finding was in agreement with previous studies of patients with acute viral gastroenteritis [20–22].

In the current study, the most common virus detected was rotavirus (39/100). This finding was in line with previous reports from Egypt (31%) and other geographical regions on children below five years old [23–25]. The global rotavirus surveillance network established by the World Health Organization estimated that the annual mortality in children below five years due to rotavirus is approximately equal to 215,000 worldwide [26]. There are two live attenuated virus vaccines for rotavirus with licenses in more than 100 countries worldwide since 2006 [27]. However, even in the post-vaccination era, the rotavirus remains a common infecting virus [28, 29]. It is noteworthy that Rotavirus vaccination is not scheduled as a routine vaccination program in Egypt. Additionally, the vaccines were less effective in African children and did not cover all circulating rotavirus genotypes [30, 31].

Among the 100 studied children with AGE, norovirus and astrovirus were detected in 27% and 12%, respectively. These prevalence rates were similar to previous studies from Egypt [32], the Republic of Congo (10.3%) [33], and India (12.5%) [34]. Nevertheless, the rates were higher than reported in a previous study from Kenya (6.3%) [35]. The prevalence of these viruses was lower than others obtained in Egypt (28%) and Nigeria (40.4%) [36, 37]. The variation in the prevalence rates can be attributed to the difference between geographical regions and the difference in socioeconomic factors [32].

RT-PCR is a sensitive method for detecting viral RNA in the stool samples of children with AGE. However, the clinicians usually limit the laboratory diagnosis of viral pathogens only to children with mild-to-moderate AGE illness because AGE treatment is supportive care. Therefore, there is limited data about the prevalence of viruses in children with AGE [38].

The association of human parechoviruses with AGE has been studied in various geographical regions such as Asia, Europe, and the Americas [39–41]. However, there is no report about this virus being associated with AGE in Egypt to the best of our knowledge. HPeV RNA was detected in 19/100 of the studied stool samples. There were different prevalence rates for HPeV in different studies. The prevalence ranged from 2.3% up to 55% [39, 42–44].

RT-PCR detected human pparechoviruses as a single pathogen in five (5%) children with AGE. Also, HPeV was detected as a single virus in 14.6% of Thai children [45]. In South Korea, 348 samples of gastroenteritis patients were tested, and only 2% turned out to be positive for human HPeV genotypes 1 and 4 [46]. Similarly, 8.1% of Japanese children, negative for other viruses, had HPeV-1 and three infections [47].

There was a debate whether HPeV as a single virus could be linked to AGE as a previous study in Germany that included 538 samples from AGE children and control samples from children without enteritis demonstrated an insignificant association between HPeV and AGE [41]. A similar finding was also reported in a study in China [39]. Therefore, a large case–control study is needed to clarify if the HPeV could be a single causative agent of AGE in children.

The comparison of the prevalence of HPeV among various studies might differ according to the demographic data of the included patients, the geographic location, and the detection method [9].

In the present study, the majority (14/19, 73.7%) of the detected HPeV was associated with other investigated enteric viruses. It was detected in association with rotavirus in nine samples (43.9%), norovirus in seven samples (36.8%), and astrovirus in two samples (10.5%); this finding was similar to previous reports [39, 40, 48].

The presenting clinical symptoms of AGE associated with HPeV might reflect the viral load. A previous report from China revealed that the genotypes of the infecting HPeV and the load of the virus could correlate with the severity of diarrhea in children with AGE [44]. In the present study, AGE caused only by HPeV was mildly severe in 60% of the infected case.

Limitations of the present study included the absence of genotyping of the detected HPeV and the non-inclusion of control children to evaluate the role of HPeV as a pathogen associated with AGE.

## Conclusion

The present study highlights that HPeV is not a rare cause of AGE among Egyptian children under five years old, with an overall detection rate of 19%. The prevalence rate of the HPeV as a single viral pathogen of AGE was 5%. Future studies are needed on a larger sample size with genotyping of the HPeV to identify the most prevalent genotype in Egypt.

## Data Availability

The data of the present study is available at https://zenodo.org/record/6125228#.Yg5yWehBzcs.

## References

[1] Raboni SM, Damasio GAC, Ferreira CE (2014). Acute gastroenteritis and enteric viruses in hospitalized children in southern Brazil: aetiology, seasonality and clinical outcomes. Mem Inst Oswaldo Cruz.

[2] Elliott EJ (2007). Acute gastroenteritis in children. BMJ.

[3] Clark B, McKendrick M (2004). A review of viral gastroenteritis. Curr Opin Infect Dis.

[4] Thongprachum A, Khamrin P, Pham NTK (2017). Multiplex RT-PCR for rapidly detecting viruses commonly causing diarrhea in pediatric patients. J Med Virol.

[5] Cai XY, Wang Q, Lin GY, Cai ZW, Lin CX, Chen PZ (2014). Respiratory virus infections among children in South China. J Med Virol.

[6] de Crom SC, Rossen JW, van Furth AM, Obihara CC (2016). Enterovirus and (HPeV) infection in children: a brief overview. Eur J Pediatr.

[7] Cordey S, L’Huillier AG, Turin L, Gervaix A, Posfay Barbe K, Kaiser L (2015). Enterovirus and *(HPeV) viremia* in young children presenting to the emergency room: unrecognised and frequent. J Clin Virol.

[8] Shakeel S, Dykeman EC, White SJ, Ora A, Cockburn JJB, Butcher SJ (2017). Genomic RNA folding mediates assembly of human (HPeV). Nat Commun.

[9] Olijve L, Jennings L, Walls T (2018). Human (HPeV): an increasingly recognized cause of sepsis-like illness in young infants. Clin Microbiol Rev.

[10] Noel JS, Lee TW, Kurtz JB, Glass RI, Monroe SS (1995). Typing of human astroviruses from clinical isolates by enzyme immunoassay and nucleotide sequencing. J Clin Microbiol.

[11] Wildenbeest JG, Benschop KSM, Bouma-De Jongh S, Wolthers KC, Pajkrt D (2016). Prolonged shedding of human (HPeV) in feces of young children after symptomatic infection. Pediatr Infect Dis J..

[12] Phan TG, Nguyen TA, Shimizu H (2005). Identification of enteroviral infection among infant and children admitted to hospital with acute gastroenteritis in Ho Chi Minh City, Vietnam. J Med Virol.

[13] Joki-Korpela P, Hyypia T (1998). Diagnosis and epidemiology of echovirus 22 infections. Clin Infect Dis.

[14] Al-Sunaidi M, Williams CH, Hughes PJ, Schnurr DP, Stanway G (2007). Analysis of a new human (HPeV) allows the definition of (HPeV) types and the Identification of RNA structural domains. J Virol.

[15] Benschop KS, Schinkel J, Luken ME, van den Broek PJ, Beersma MF, Menelik N (2006). Fourth human (HPeV) serotype. Emerg Infect Dis.

[16] Rizk NM, Abd-Elmaksoud S, Farid TM, Abohashish MMA, Al-Herrawy AZ, Hamza IA (2021). Etiology of diarrheal disease among children under 5 years in Egypt: a high incidence of human bocavirus. J Egypt Public Health Assoc.

[17] Zaki ME, NermenKheir AE (2017). Molecular study of astrovirus, adenovirus and norovirus in community-acquired diarrhea in children: one Egyptian center study. Asian Pac J Trop Biom..

[18] Vesikari KL. Clinical Severity Scoring manual. 2011.

[19] Chen BC, Cheng MF, Huang TS, Liu YC, Tang CW, Chen CS, Chen YS (2009). Detection and identification of human (HPeV)es from clinical specimens. Diagn Microbiol Infect Dis.

[20] Lu L, Zhong H, Xu M (2021). Molecular and epidemiological characterization of human adenovirus and classic human astrovirus in children with acute diarrhea in Shanghai, 2017–2018. BMC Infect Dis.

[21] Kim JS, Lee WJ, Lee SK, Lee EJ, Hyun J, Kim HS (2019). Molecular epidemiology of human astrovirus in stool samples from patients with acute gastroenteritis in Korea, 2013–2017. Ann Lab Med.

[22] Sharif N, Parvez AK, Haque A, Talukder AA, Ushijima H, Dey SK (2020). Molecular and epidemiological trends of human bocavirus and adenovirus in children with acute gastroenteritis in Bangladesh during 2015 to 2019. J Med Virol.

[23] Kamal Allayeh A, Mostafa El-Baz R, Mohamed Saeed N, El Sayed OM (2018). Detection and genotyping of viral gastroenteritis in hospitalized children below five years old in Cairo, Egypt. Arch Pediatr Infect Dis.

[24] Jeff DO, Aziz TAG, Smith NR (2015). The incidence of rotavirus and adenovirus infections among children with diarrhea in Sulaimani Province, Iraq. J Biosci Med.

[25] Tran A, Talmud D, Lejeune B, Jovenin N, Renois F, Payan C, Nicolas Leveque N, Andreoletti L (2010). Prevalence of rotavirus, adenovirus, norovirus, and astrovirus infections and co-infections among hospitalized children in northern France. J Clin Microbiol.

[26] Tate JE, Burton AH, Boschi-Pinto C, Steele AD, Duque J, Parashar UD (2016). Global, regional, and national estimates of rotavirus mortality in children %3c 5 years of age, 2000–2013. Clin Infect Dis.

[27] Parashar UD, Johnson H, Steele AD, Tate JE (2016). Health impact of rotavirus vaccination in developing countries: progress and way forward. Clin Infect Dis.

[28] Ahmed SM, Hall AJ, Robinson AE (2014). Global prevalence of norovirus in cases of gastroenteritis: a systematic review and meta-analysis. Lancet Infect Dis.

[29] O’Ryan ML, Peña A, Vergara R (2010). Prospective characterization of norovirus compared with rotavirus acute diarrhea episodes in Chilean children. Pediatr Infect Dis J.

[30] Madhi SA, Cunliffe NA, Steele D, Witte D, Kirsten M, Louw C (2010). Effect of human rotavirus vaccine on severe diarrhea in African infants. N Engl J Med.

[31] Harris VC, Armah G, Fuentes S, Korpela KE, Parashar U, Victor JC (2017). Significant correlation between the infant gut microbiome and rotavirus vaccine response in rural Ghana. J Infect Dis.

[32] El Sayed ZM, Mashaly GE, Alsayed MAL, Nomir MM (2020). Molecular study of human astrovirus in Egyptian children with acute gastroenteritis. Germs.

[33] Nguekeng Tsague B, Mikounou Louya V, Ntoumi F (2020). Occurrence of human astrovirus associated with gastroenteritis among Congolese children in Brazzaville, Republic of Congo. Int J Infect Dis.

[34] Akdag AI, Gupta S, Khan N, Upadhayay A, Ray P (2019). Epidemiology and clinical features of rotavirus, adenovirus, and astrovirus infections and co-infections in children with acute gastroenteritis prior to rotavirus vaccine introduction in Meerut, North India. J Med Virol.

[35] Kiulia NM, Mwenda JM, Nyachieo A, Nyaundi JK, Steele AD, Taylor MB (2007). Astrovirus infection in young Kenyan children with diarrhea. J Trop Pediatr.

[36] El Taweel A, Kandeil A, Barakat A, Alfaroq Rabiee O, Kayali G, Ali MA (2020). Diversity of astroviruses circulating in humans, bats, and wild birds in Egypt. Viruses.

[37] Ayolabi C, Ojo D, Akpan I (2012). Astrovirus infection in children in Lagos, Nigeria. Afr J Infect Dis.

[38] Halasa N, Piya B, Stewart LS (2021). The changing landscape of pediatric viral enteropathogens in the post-rotavirus vaccine era. Clin Infect Dis.

[39] Zhang DL, Jin Y, Li DD, Cheng WX, Xu ZQ, Yu JM, Jin M, Yang SH, Zhang Q, Cui SX (2011). prevalence of human (HPeV) in Chinese children hospitalized for acute gastroenteritis. Clin Microbiol Infect.

[40] Pham NT, Takanashi S, Tran DN, Trinh QD, Abeysekera C, Abeygunawardene A, Khamrin P, Okitsu S, Shimizu H, Mizuguchi M (2011). Human (HPeV) infection in children hospitalized with acute gastroenteritis in Sri Lanka. J Clin Microbiol.

[41] Baumgarte S, de Souza Luna LK, Grywna K, Panning M, Drexler JF, Karsten C, Huppertz HI, Drosten C (2008). Prevalence, types, and RNA concentrations of human (HPeV)es, including a sixth (HPeV) type, in stool samples from patients with acute enteritis. J Clin Microbiol.

[42] Seo JH, Yeom JS, Youn HS, Han TH, Chung JY (2015). Prevalence of human (HPeV) and enterovirus in cerebrospinal fluid samples in children in Jinju, Korea. Korean J Pediatr.

[43] Zhong H, Lin Y, Sun J, Su L, Cao L, Yang Y, Xu J (2011). Prevalence and genotypes of human (HPeV) in stool samples from hospitalized children in Shanghai, China, 2008 and 2009. J Med Virol.

[44] Zhang XA, Zhao RQ, Chen JJ, Yuan Y, Tang X, Zhou ZW, Ren L, Lu QB, Wang YN, Zhang HY, Zhang PH, Fang LQ, Zhou HS, Liu EM, Xu HM, Liu W (2021). The identification and genetic characterization of (HPeV) infection among pediatric patients with wide clinical spectrum in Chongqing. China Front Microbiol.

[45] Pham NT, Trinh QD, Khamrin P, Maneekarn N, Shimizu H, Okitsu S, Mizuguchi M, Ushijima H (2010). Diversity of human (HPeV)es isolated from stool samples collected from Thai children with acute gastroenteritis. J Clin Microbiol.

[46] Han TH, Kim CH, Park SH, Chung JY, Hwang ES (2011). Detection of human (HPeV)es in children with gastroenteritis in South Korea. Arch Virol.

[47] Pham NT, Chan-It W, Khamrin P, Nishimura S, Kikuta H, Sugita K, Baba T, Yamamoto A, Shimizu H, Okitsu S (2011). detection of human (HPeV) in stool samples collected from children with acute gastroenteritis in Japan during 2007–2008. J Med Virol.

[48] Guo Y, Duan Z, Qian Y (2013). Changes in human (HPeV) profiles in hospitalized children with acute gastroenteritis after a three-year interval in Lanzhou, China. PLoS ONE.

